# Supplementary material for: Wave-induced loss of ultra-relativistic electrons in the Van Allen radiation belts
Source: Nat Commun. 2016 Sep 28;7:12883. doi: 10.1038/ncomms12883 (PMC5052794; doi:10.1038/ncomms12883)
Supplement: Supplementary Information — Supplementary Figures 1-6, Supplementary Table 1, Supplementary Notes 1-6 and Supplementary References [file ncomms12883-s1.pdf]

Supplementary Figures

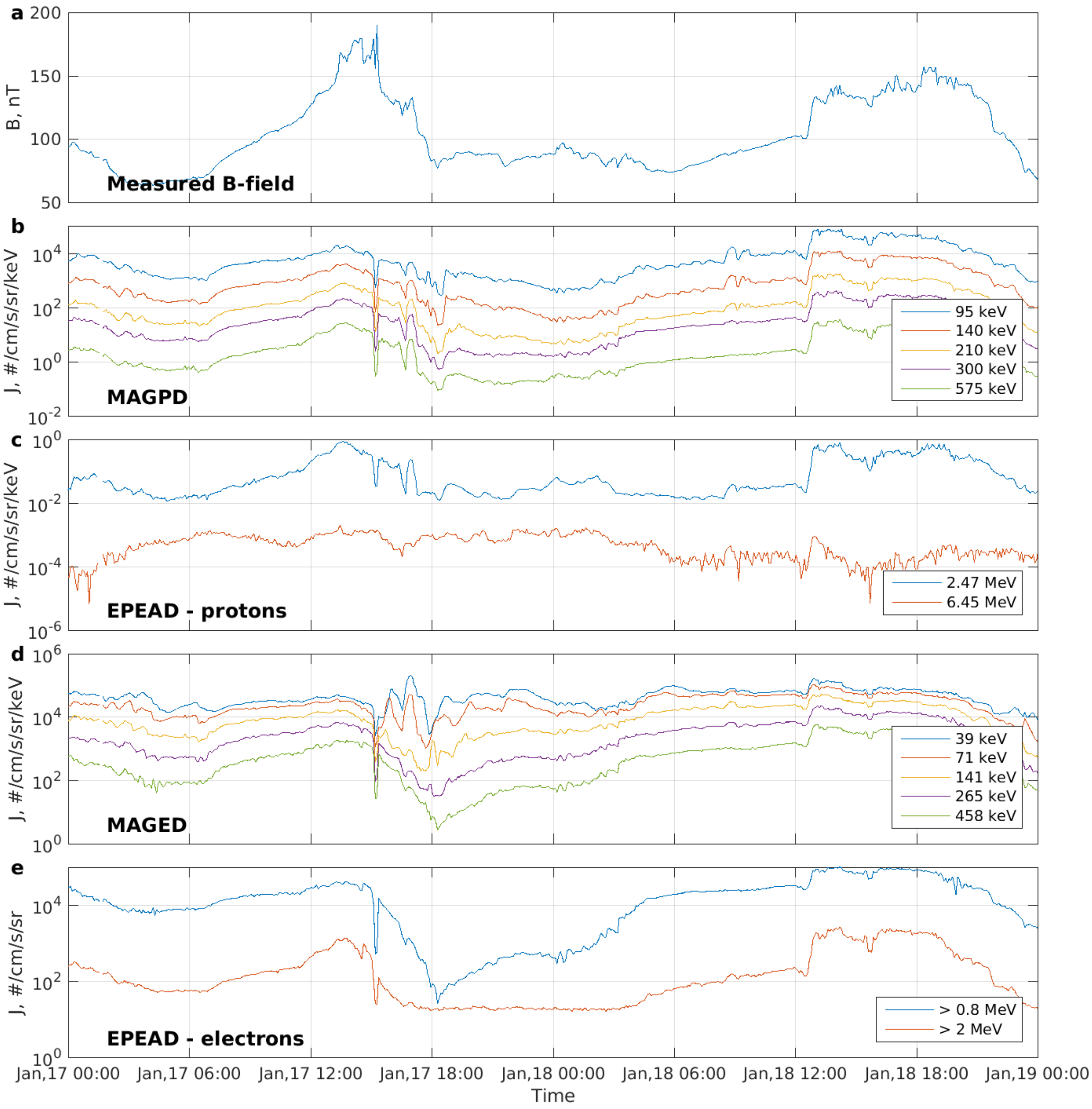

Supplementary Figure 1 GOES observations of a) total magnetic field, b) differential proton fluxes from MAGPD, c) differential proton fluxes from EPEAD, d) differential electron fluxes from MAGED, and e) integral electron fluxes from EPEAD. A similar variation in electron and proton fluxes are observed over the January 17th to January 19th interval shown across keV to MeV energies. Note that 2 MeV reached the instrumental noise floor level which explains flat plateau from ~1700 on Jan 17th until 4am on Jan 18th. Ultra-relativistic energy electrons are not available on GOES.

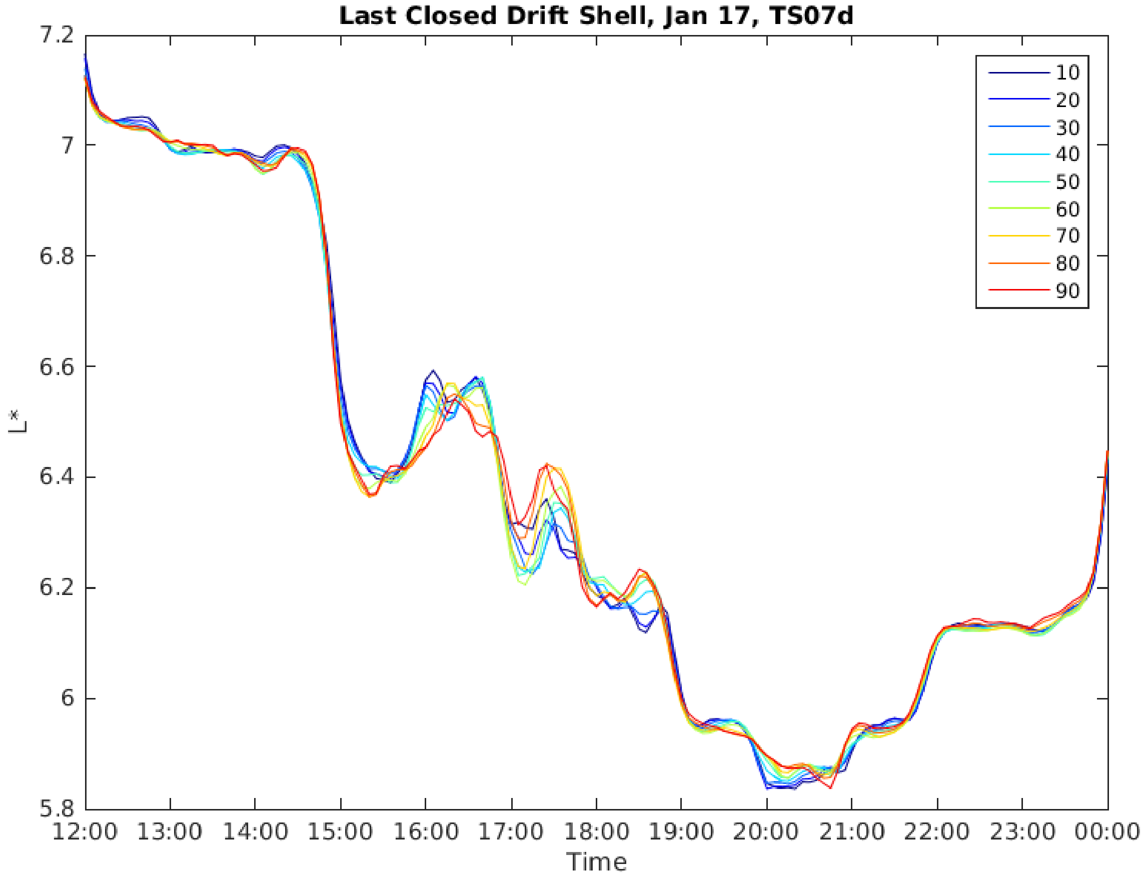

Supplementary Figure 2. **Location of the last closed drift shell (L*).** The location of the last closed drift-shell calculated in the TS07d magnetic field model (mid 2015 coefficients) and given as a function of time for equatorial pitch angles from 10 to 90 degrees.

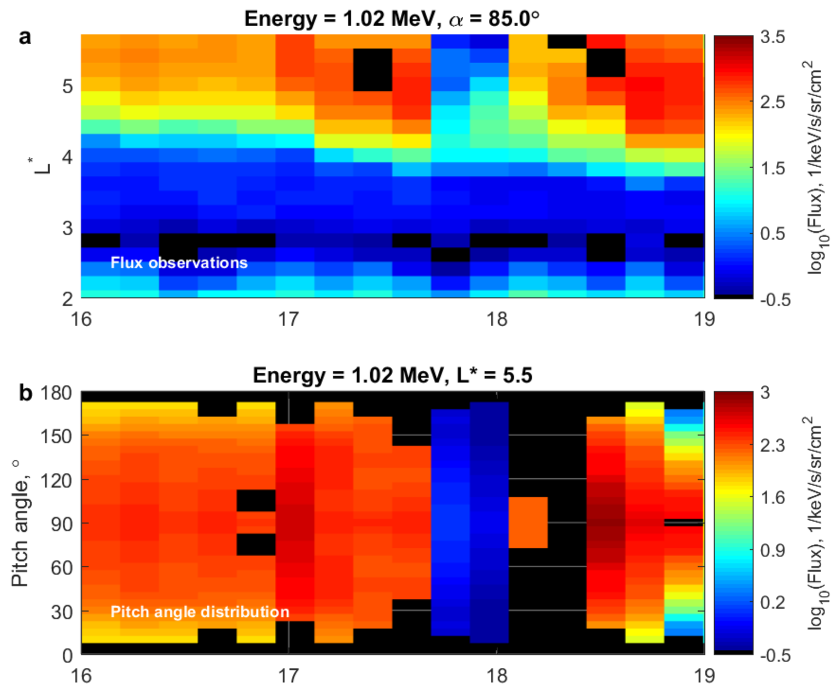

Supplementary Figure 3. Measurements of 1.02 MeV electron fluxes and local pitch angle distribution. a) Observation of electron fluxes at 85° local pitch angle, b) local pitch angles distribution at L*=5.5

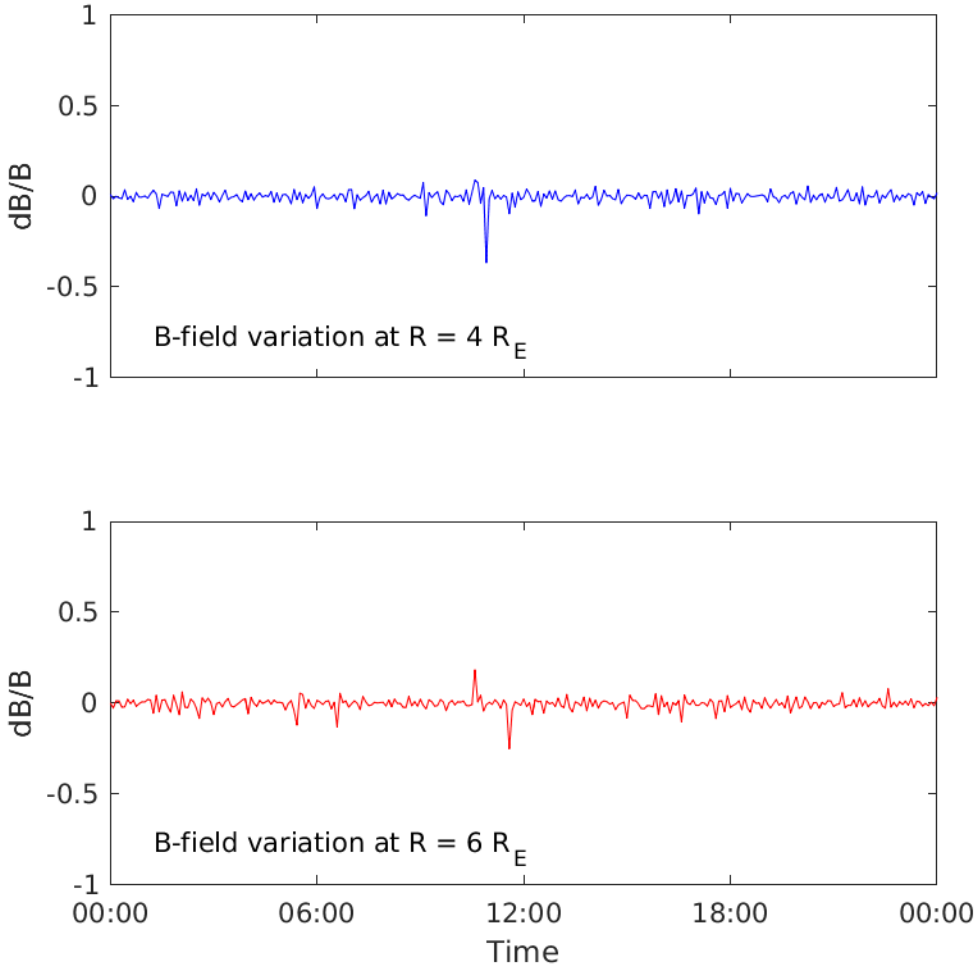

Supplementary Figure 4. The change in B-field normalized by the B-field (ΔB/B) variation
Calculations were done in the TS07D B-field model was computed for several locations in the inner magnetosphere and on a five-minute cadence on the 17th of January 2013. The ΔB/B variation is averaged across all MLT sectors, and displayed here for R = 4 Re in blue, and R = 6 Re in red. There is a brief period pre-noon on the 17th where the variation is ~35% of the modeled B-field, otherwise it is below 10% of modeled B for the remainder of the period.

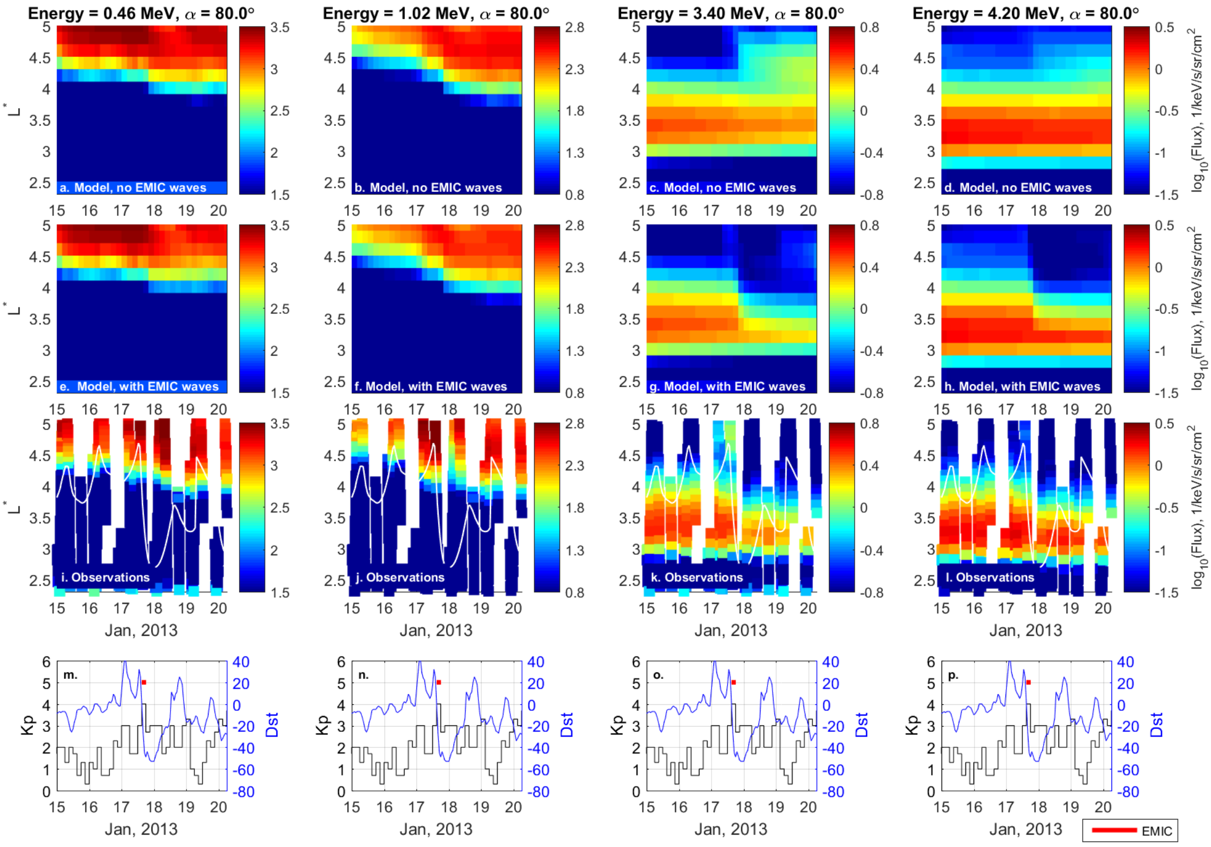

Supplementary Figure 5.Comparisons of the evolutions of the modeled and observed radial profiles of fluxes. Similar to Figure 3, but the data are not orbit averaged and the center of each pixel corresponds to the exact time of the measurement. Energies of 0.46, 1.02, 3.40, 4.20 MeV are presented.

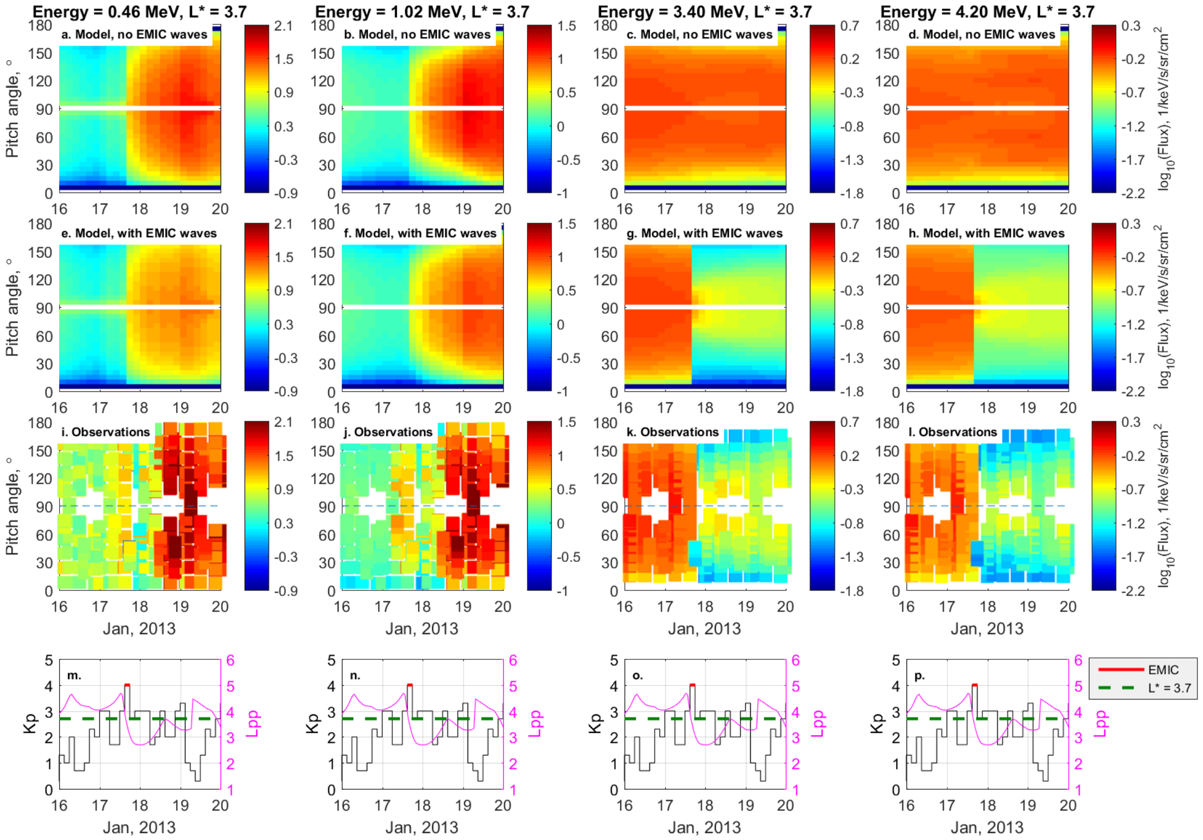

Supplementary Figure 6. **Evolution of the pitch angle distribution during the January 17, 2013 storm**. Similar to Figure 4, but the data are not orbit averaged and the center of each pixel corresponds to the exact time of the measurement. Energies of 0.46, 1.02, 3.40, 4.20 MeV are presented.

Supplementary Tables

Supplementary Table 1. Spectral parameters of the EMIC waves diffusion coefficients

Amplitude, nT	Central frequency, Ω_{O^+}	Frequency bandwidth, Ω_{O^+}	Lower cutoff frequencies, Ω_{O^+}	Upper cutoff frequencies,Ω_{O^+}
0.90	3.24	0.25	3.60	3.00
0.30	3.24	0.02	3.60	3.00
0.30	3.72	0.20	3.84	3.60

Supplementary Notes

Supplementary Note 1. Temporal variations at 1 MeV

The apparent temporal dropout of fluxes on January 17th is mostly adiabatic. First of all fluxes promptly return to pre-storm values which is indicative of adiabatic change. If EMIC waves were responsible for the losses at 1 MeV then we should expect a narrowing pitch angle distribution, as observed for ultra-relativistic electrons. On the contrary, a broadening pitch angle distribution was observed (see Figure 4e). Furthermore, a very similar adiabatic dropout in electrons occurs at lower energies that certainly cannot be affected by EMIC waves. Observations on GOES clearly confirm that (See Supplementary Figure 1) Electrons and Ions at all energies drop simultaneously when magnetic filed decreases and recover to prestorm values when the magnetic field increases back. This time period corresponds to the temporal dropout on Figure 1c. Note that higher multi-MeV electrons that are shown in the main part of the manuscript do not recover to the prestorm values.

Supplementary Note 2. Losses to the magnetopause

This note provides additional evidence that the ultra-relativistic dropout is not produced by the loss to the magnetopause. Near 15 UT, on the 17th January, 2013, the last closed drift shell in the TS07d magnetic field model was near L* = 6.4. The minimum L* value evolution for 10 - 90 degree equatorial pitch angle electrons is shown in Supplementary Figure 2. While this L-value is lower than magnetopause location it is still above the upper boundary of the simulation. Since the magnetopause does not cross the upper boundary in radial distance no additional loss needs to be included in the simulation. Radial profiles of simulations without EMIC waves included (Figure 3b) clearly show that fluxes of ultra-relativistic electrons increase. That means that the outward radial diffusion driven by the loss to the magnetosphere does not contribute to the observed dropout at multi-MeV energies.

Additional evidence of EMIC wave scattering comes from the observations of pitch angle distributions. At 90 degree pitch angle, particles are more likely to be lost to the magnetopause, and so butterfly-shaped pitch angle distributions in the flux observations are indicative of the loss to the magnetopause. Observations at L*=5 (see Supplementary Figure 3) show that even at higher L-shells pitch angle distributions did not exhibit a butterfly distribution, which indicates that loss to the magnetopause did not significantly propagate down to L=5 and below. If losses near L*=5 were not significant, then one certainly would not expect the magnetopause to contribute to the observed losses at L* = 3- 4, where ultra-relativistic belt is located. Even if the loss to the magnetopause contributes to the loss of MeV electrons at higher L-shells that does not affect the conclusions of this study, as ultra-relativistic electron dropout occurs below L=4.

Supplementary Note 3, On global B-field variations in the inner magnetosphere during the study period.

Using the TS07D magnetic field model[3] the change in B-field normalized by the B-field ($\Delta B/B$) was computed on a 5-minute cadence for several radial distances and MLT locations from R = 3-6 Re on the 17th January, 2013. The averaged $\Delta B/B$ for two radial distances, R = 4 and R = 6, are shown by

blue and red lines, respectively, in Supplementary Figure4. The only substantial change in B was a ~35% variation at pre-noon, 3 hours before the observed EMIC wave interval of 15-18 UT. For the remainder of the period no variation exceeding 10 % of modeled B occurred, and in fact the variation was typically much lower. For R=4 variations are below 20 % and cannot explain the bite outs at relativistic energies or difference in the dynamics of relativistic and ultra-relativistic electrons. Satellite measurements indicate that variations of the field observed on Van Allen Probes are even smaller than predicted by the TS07D model. Since no significant variation occurred during the time where the loss of multi-MeV electrons and narrowing of the pitch angle distribution was observed, the global B-field variation is not responsible for the observed losses and narrowing pitch angle distribution presented in this study.

Supplementary Note 4, On interpretation of ground observations

For specific event studies, satellite observations may not be available at locations where waves are present. Often ground-based observations of the EMIC waves are used since there exists a multitude of magnetometer arrays spread in longitude about the globe. However, due to various wave propagation effects such as ionospheric ducting, EMIC waves that originate near the equatorial plane at a single L shell may be observed on the ground over a much wider range of L. It is also useful to estimate the proton, helium and oxygen cyclotron frequencies in the equatorial magnetosphere and compare these frequencies to the observed frequencies of the waves.

Waves observed at the surrounding stations have a similar frequency time structure but lower intensity. Ground observations cannot provide us exact range of radial distances but it's likely that the peak intensity was near 4.5 R_E. Due to propagation effects the EMIC waves may be observed at both the higher and lower latitude stations, which explains the signal observed at the station located at L=3.4.

This is also confirmed by examining the helium cyclotron frequency (white) and the oxygen cyclotron frequency (magenta) calculated by tracing the field line to the magnetic equator using the T04s model and prevailing solar wind and geomagnetic conditions as shown in Figure 2. The proton cyclotron frequency is above the limits of the plot for all panels. Here we see that only at Oulu do the wave fall distinctly into the helium band between the two cyclotron frequencies. Waves observed at Nurmijarvi map to below the helium band and likely were not generated at these low L values.

It should be also noted that waves may be present at other MLT which would be impossible to infer with a single chain of magnetometers. The presented observations clearly show that the waves were present during the modeled period of time.

Supplementary note 5. The EMIC wave properties. Spectrum

The helium band EMIC waves with a various intensities were included in the simulation on January 17, 2013, from 15:00 to 18:00.

For the calculation of the diffusion coefficients the maximum latitudinal distribution of waves was assumed to be 45°. The magnetospheric plasma composition was assumed to be 70% H+ , 20% He+ , and 10% O+. The chosen intensity is consistent with statistical studies [1,2]. Hiss in the region of plume and inside the plasmasphere and chorus waves are also included into the simulation. Hiss and chorus

waves are assisting EMIC waves by transporting particles from high pitch angles to small pitch angles.

Supplementary Note 6. Data processing

The TS07D[3] model was used to compute the L* and the value of the equatorial pitch angle for every point of measurement. To plot the electron fluxes that are presented on Figure 1 (c and d) and on Figure 3 (e and f) data was interpolated to 85° equatorial pitch angle. When measurements above 85° are not available we extrapolated data by using sin function. Then, the data from the satellites RBSP-A and RBSP-B were binned in the 0.2 Re L* intervals corresponding to the simulation grid points. The data were binned in time by orbital period. To obtain initial PSD profiles, the processed fluxes were converted into PSD and interpolated on to the simulation grid.

In Supplementary Figure 5 (i-l) the data were not interpolated or extrapolated, but every point of the flux measurements are shown. The data were used only in the 80°±10° window of the equatorial pitch angles for individual spacecraft. If there was more than one point in the selected window, the average was used for plotting. If the data was not available we did not extrapolate measurements to higher pitch angles. The visible gaps correspond to the times when the spacecraft were located sufficiently far from the geomagnetic equator and could not observe the electrons above 70° of the equatorial pitch angle. The evolution of fluxes is similar to the evolution shown on Figure 3 indicating that binning, interpolation or extrapolation does not change the dynamics of fluxes. We also presented on the Supplementary Figure 1 two more energy channels that support the conclusions of the main part of this study.

Figure 1(e and f), Figure 4(e and f) and Supplementary Figure 6(i-l) show the pitch angle distribution for different energies at L*=3.9. The data were binned in L* with a 0.2 R_E step. Data was not interpolated or extrapolated in pitch angle. Note that Figure 1(e and f) and Figure 2(e and f) show orbit averaged while Supplementary Figure 6(i-l) shows pixels centered at the times when measurements occurred.

The size of the pixels for Supplementary Figure 6(i-l) Supplementary Figure 6(i-l) is chosen to be large enough to show clear evolution. The gaps around 90° show the absence of observation at the high equatorial pitch angles.

Supplementary References

1. Meredith, N. P., Horne, R. B., Kersten, T., Fraser, B. J. & Grew, R. S. Global morphology and spectral properties of EMIC waves derived from CRRES observations. *J. Geophys. Res. [Space Phys]* **119,** 5328–5342 (2014).
2. Saikin, A. A. *et al.* The occurrence and wave properties of H+-, He+-, and O+-band EMIC waves observed by the Van Allen Probes. *J. Geophys. Res. [Space Phys]* **120,** 2015JA021358 (2015).

3. Tsyganenko, N. A. & Sitnov, M. I. Magnetospheric configurations from a high-resolution data-based magnetic field model. *J. Geophys. Res.* **112,** A06225 (2007).
